# Comparative Efficacy of Low-Level Laser Acupuncture and Electroacupuncture in Women With Dysmenorrhea and Autonomic Imbalance: A Pilot Randomized-Controlled Trial

**DOI:** 10.1155/prm/3494216

**Published:** 2025-10-23

**Authors:** Tsai-Ju Chien, Yuan-I. Chang, Li-Lan Liao, Yu-Ni Hsu, Chien-Wen Huang

**Affiliations:** ^1^Division of Hemato-Oncology, Department of Internal Medicine, Taipei City Hospital, Branch of Zhong-Xing, Taipei, Taiwan; ^2^Institute of Traditional Medicine, National Yang-Ming Chiao Tung University, Taipei, Taiwan; ^3^Department and Institute of Physiology, College of Medicine, National Yang Ming Chiao Tung University, Taipei, Taiwan; ^4^Department of Traditional Medicine, Taipei City Hospital, Branch of Kunming, Taipei, Taiwan; ^5^Sunhawk Vision Biotech, Inc., Taipei, Taiwan

**Keywords:** autonomic dysfunction, dysmenorrhea, electroacupuncture, heart rate variation, low-level laser acupuncture

## Abstract

**Background:**

Electroacupuncture (EA) has been widely applied in treating dysmenorrhea and autonomic dysfunction. Low-level laser acupuncture (LLLA), a noninvasive acupuncture, has been used clinically, but efficacy is uncertain. This study aimed to explore the efficacy of LLLA, EA, and sham-controlled LLLA on pain, heart rate variability (HRV) activity, and symptom improvement in dysmenorrhea with autonomic imbalance.

**Method:**

In a randomized, controlled design, a total of 114 women with dysmenorrhea and autonomic imbalance were randomly allocated to three groups: sham LLLA, LLLA, and EA. The primary outcomes are pain (VAS), and HRV parameters, and the secondary outcomes are symptom assessment (verbal multidimensional scoring system), prostaglandin E, progesterone levels, and the SF-12 quality-of-life assessment.

**Results:**

Contrast to the sham acupuncture, LLLA and EA effectively reconciled the pain and sympathetic/parasympathetic tone significantly, no matter the vagus or sympathetic deviated status (low or high LF/HF group). LLLA was superior to EA in terms of SDNN, RMMSD, and PNN50 (*p* < 0.001) in the low LF/HF group in intergroup analysis. For all participants' analysis, the effects of LLLA and EA were similar for pain assessment (VAS) and quality of life (SF-12) (*p* < 0.05). In proinflammatory cytokine measurement, EA showed a significant decrease in prostaglandin E2 (PGE2) levels (*p* = 0.022).

**Conclusion:**

This pilot study showed both LLLA and EA effectively relieve dysmenorrhea without a placebo effect, LLLA is noninferior to EA in autonomic dysfunction, and the effect is even more prominent in low LF/HF status (a relatively lower energy status). Given its noninvasiveness, LLLA could be an alternative treatment and warrants further large-scale study.

**Trial Registration:**

ClinicalTrials.gov identifier: NCT04178226

**Summary**

1. Laser acupuncture is noninferior to electroacupuncture (EA) in dysmenorrhea.
2. Novelty in analyzing acupuncture effect by ANS imbalance (vagus or sympathetic predominant status by applying LF/HF ratio).
3. Updated RCT with sham controlled for laser acupuncture in dysmenorrhea.
4. EA significantly decreases prostaglandin levels in dysmenorrhea.
5. Laser acupuncture is superior to EA, especially in low LF/HF status.

## 1. Introduction

Primary dysmenorrhea (PD) is a prevalent gynecological disorder among women of reproductive age, with incidence rates reported between 50% and 65% [1]. While nonsteroidal anti-inflammatory drugs (NSAIDs) and hormonal therapies are standard treatments, their limitations and adverse effects have prompted increasing interest in alternative modalities such as acupuncture [2, 3]. The analgesic efficacy of acupuncture in dysmenorrhea has been substantiated in multiple clinical trials [4, 5], and its mechanisms are thought to involve modulation of the autonomic nervous system (ANS) and neuroendocrine pathways. Low-level laser acupuncture (LLLA), as a noninvasive alternative, has also been applied in clinical settings, such as neuropathy [6, 7], dentistry [6], arthritis [8], chronic pain [9], and many other fields. Accumulating evidence from clinical trials has demonstrated the efficacy of acupuncture in alleviating dysmenorrhea, with mechanisms believed to involve modulation of the ANS [10, 11] and neuroendocrine pathways [12, 13]. However, its comparative efficacy relative to electroacupuncture (EA) remains uncertain. Notably, both LLLA and EA have been investigated for their potential to regulate autonomic function and alleviate pain in dysmenorrhea [14], yet direct comparative studies remain limited. To address this issue, our team has published a crossover study directly comparing LLLA and EA in dysmenorrhea, which supports the potential of LLLA [15].

The prior study we published in *JICM* utilized a crossover design, ensuring that all participants receive both interventions and thereby adhering to ethical standards by providing equitable access to all treatments [15]. Nonetheless, the lack of a control group in previous studies has raised concerns regarding potential bias in the assessment of acupuncture-related outcomes. Prior research has underscored the importance of incorporating sham acupuncture controls to enhance the methodological rigor and validity of such investigations. To address these limitations, the current study adopts a three-arm design that includes a sham-controlled group, enabling a robust comparison of the therapeutic efficacy of LLLA and EA relative to sham acupuncture. This methodological approach is intended to improve the reliability and interpretability of the findings. Moreover, the study specifically targets patient subgroups characterized by autonomic imbalance, defined by either sympathetic or parasympathetic predominance, and builds upon our previous research.

## 2. Materials and Methods

### 2.1. Study Design

This pilot study employed a randomized, placebo-controlled, partially double-blinded clinical trial to rigorously compare the therapeutic efficacy of LLLA and EA with that of sham LLLA in dysmenorrhea (VAS > 3). As our primary focus was to compare the efficacy of noninvasive LLLA (with sham control) and invasive EA, implementing a sham-EA group posed significant practical and ethical challenges, including increased discomfort and technical limitations of blinding needle interventions. Therefore, a three-arm trial has been eventually adopted.

This methodological approach enhances the reliability and interpretability of findings, particularly in subgroups characterized by autonomic imbalance (either sympathetic or parasympathetic predominance; normal LF/HF as a useful index of autonomic interactive modulation ranging from 0.8 to 1.2 [16, 17]).

### 2.2. Ethics and Participants

The study was approved by the Human Ethics Committee of Taipei City Hospital (IRB no. 10712112) and conducted at the Traditional Chinese Medicine (TCM) outpatient department, Taipei City Hospital. The protocol was registered with ClinicalTrials.gov The 2013 revision of the Helsinki Declaration and the ethical rules of our hospital's Ethics Committee were followed. Each participant signed an informed consent form.

**Figure 1:**
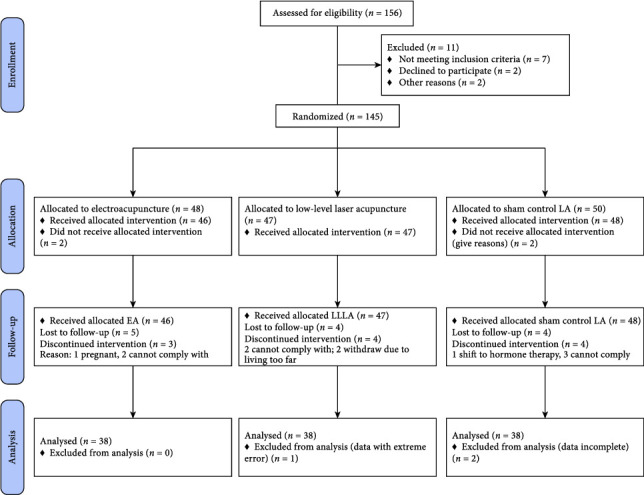
Study flow chart (CONSORT 2010).

Eligible participants were women aged 15–49 years with PD (at least grade II) and autonomic imbalance, defined by LF/HF ratios outside the range of 0.8–1.2. Exclusion criteria included dysmenorrhea due to intrauterine devices, mild or occasional dysmenorrhea, suspected malignancy, or psychiatric disorders potentially affecting autonomic function. Written informed consent will be obtained from all participating patients before randomization. Participants were randomly assigned to receive LLLA, EA, or sham LLLA, with all interventions administered at standardized acupoints and frequency. Baseline demographic and clinical characteristics were balanced among groups. The CONSORT flow diagram of study sample selection and assessment schedule is shown in Figure 1.

### 2.3. Randomization, Allocation, and Blinding Method

Participants were randomized using an online randomizer (https://www.randomizer.org) into three groups: LLLA, EA, and sham LLLA control (https://www.randomizer.org).

By using the tool, participants might receive codes “Group A,” “Group B,” and “Group C” without revealing the treatment assignments. All participants only knew that they would receive one of three types of acupuncture treatment and were only informed of their group code. The study is partially double-blinded. Due to the distinct differences in intervention procedures, complete blinding of participants is not feasible in this study. Consequently, a partial double-blind design is implemented: on one hand, for the laser acupuncture versus sham laser acupuncture, blinding is generally effective here. In this study, participants cannot reliably distinguish between active and sham laser acupuncture, as both procedures look and feel similar, and the sham device emits visible light but no therapeutic laser energy. This makes sham LA a credible placebo control for LA. On the other hand, for the electro-acupuncture versus laser/sham laser acupuncture, blinding participants between EA and the two laser-based groups is not feasible because the procedures are obviously different: EA involves needle insertion and electrical stimulation, while LA and sham LA are noninvasive and do not use needles. Participants will be able to tell if they are receiving EA or a non-needle intervention.

While practitioners are necessarily aware of group allocations, the personnel responsible for data management and analysis remain blinded to participant assignment until the trial is completed. All data were anonymized from participants' enrollment to the assessment process. When collecting and labeling data, use only the group codes (e.g., Group A, B, and C) without reference to the actual intervention. This ensures that data analysts who view or process the data cannot infer which treatment group corresponds to which intervention.

### 2.4. Sample Size and G-Power Software

Sample size was calculated by the following formula:

(1) $$\begin{array}{c} n=\frac{{\left(Z_{\alpha /2}+Z_{\beta }\right)}^{2}\times 2\sigma^{2}}{{\left(\mu_{\text{EA}}-\mu_{\text{LLLA}}\right)}^{2}}, \end{array}$$

where *μ*_EA_ is the reduction of VAS score in the EA group and *μ*_LLLA_ is the reduction of VAS score in the LA group.

We selected VAS for sample size calculation because pain is the primary outcome in dysmenorrhea with autonomic imbalance. The LF/HF ratio, though relevant, is unsuitable due to interpretative variability and limited use in sample size estimation.

G-Power was applied to estimate the minimum number of participants needed per group to detect a statistically significant difference in pain reduction (VAS score) between the intervention arms (LLLA and EA), considering a desired power (typically 80%), significance level (*α* = 0.05), and the expected effect size (mean difference of 1.0 points in VAS score with standard deviation of 1.6 based on previous research [18]). By inputting these parameters into G∗Power—choosing the appropriate test type (ANOVA) and an allocation ratio of 1:1:1; a minimum of 35 participants per group was required for analysis [19, 20]. Considering the potential for a dropout rate of 30% or higher due to the impact of the COVID-19 pandemic, the target sample size was increased to 46 participants per group (totaling 138 participants). Ultimately, 145 participants were enrolled in the study, and data from 38 participants per group were included in the final analysis.

### 2.5. Interventions

The acupuncture style adopted was traditional acupuncture and laser acupuncture, and the protocol was developed following the recommendations of Standard Protocol Items: Recommendations for Interventional Trials (SPIRIT) [21]. LLLA was performed as stimulation of traditional acupoints with low-intensity, nonthermal laser irradiation [22], which was delivered by LaserPen (RJ-Laser, Reimers & Janssen GmbH, Waldkirch, Germany) with maximal power, 150 mW; wavelength, 810 nm; area of probe, 0.13 cm^2^; power density, 1.19 W/cm^2^; pulsed wave; and Nogier E frequencies; 15 s for selected acupoints delivering a total dose of 2.5 J/cm^2^ (supporting 1). Sham laser treatment was performed according to the verum treatment protocol with visual (red light: ∼587 nm) and acoustic functions in order to create a sham procedure. EA stimulation was performed after achieving the de-qi sensation, lasting 20 min with a 50 Hz continuous wave and 1–5 mA current intensity. All groups received stimulation at identical acupoints: bilateral PC6 (內関), SP4 (公孫), SP6 (三陰交), SP10 (血海), RN4 (関元), and RN6 (氣海) according to WHO guidelines [23] (supporting 2), and even the sham-controlled group received stimulation at identical acupoints yet without any laser output. All participants received treatment twice weekly during the luteal phase for three menstrual cycles (12 sessions/3 months) by trained acupuncturists. The study scheme is as supporting 3

### 2.6. Outcomes Measurement

#### 2.6.1. Primary Outcomes

The primary outcomes were pain (by VAS) and assessments of the autonomic nervous activity by measuring heart rate variability (HRV) parameters (ANSWatch, wrist monitor: Taiwan Scientific Co., Taipei, Taiwan). The measurement of HRV included the low-frequency component (LF), the high-frequency component (HF), and the LF/HF ratio (as an index for autonomic balance). We compared the pain improvement with the VAS score and also focused on the LF/HF ratio, which was specifically analyzed to distinguish between sympathetic and parasympathetic predominance, reflecting the study's focus on autonomic imbalance [17, 24]. Other parameters, such as the total HRV activity, the standard deviation of adjacent peak-to-peak intervals (SDNN), and the root mean square of the successive differences (RMSSD), one of a few time-domain tools, were also recorded to assess HRV [25]. The accuracy of the ANS monitor was represented by correlations between the HRV parameters and electrocardiogram results [26].

#### 2.6.2. Secondary Outcomes

Secondary outcomes encompassed circulating levels of prostaglandin E2 (PGE2) and progesterone. Prostaglandin has been known to have a direct relationship with dysmenorrhea [27], while lower progesterone levels are associated with increased abdominal cramps and pain symptoms, supporting a mechanistic role linking low luteal progesterone with menstrual distress [28] and clinical symptom assessments as the verbal multidimensional scoring system (VMSS) and quality of life (SF-12).

### 2.7. Statistical Analysis

Statistical analyses were performed using appropriate parametric and nonparametric tests, with adjustments for potential confounders such as age, BMI, and baseline symptom scores (IBM SPSS 22.0, Armonk, NY, USA). The within-group differences in HRV parameters, VMSS, VAS, and SF12 before and after the treatment were evaluated by paired-t test or Wilcoxon test. Between-group differences were evaluated using two-way mixed ANOVA, and multivariate regression was applied to control for confounding variables. Statistical significance was set at *p* < 0.05.

## 3. Results

### 3.1. Participants' Characteristics

A total of 145 participants were enrolled and randomized into 3 groups from August 2020 till October 2023. After exclusions, 114 patients formed the analytic sample and completed the study. Participants' demographic and clinical characteristics are depicted in Table 1. Participants' mean age was 36 years. Baseline data showed well-balanced clinical features between-groups without significant differences.

### 3.2. Comparison of Effects of EA and LLLA on Pain and Autonomic Dysfunction

We defined the LF/HF range from 0.8 to 1.2 as normal and analyzed the impact of each type of acupuncture on low LF/HF (parasympathetic dominance) or high LF/HF (sympathetic prominence), as shown in Table 2.

#### 3.2.1. In Pain Assessment

**Figure 2:**
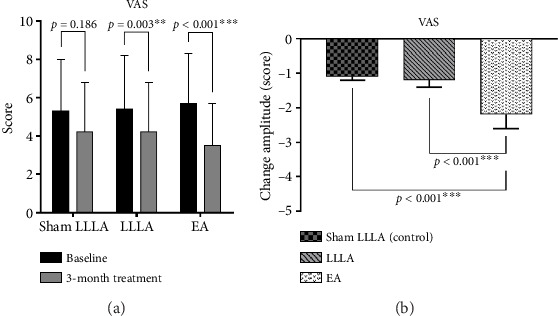
Comparison of the effect of EA and LLLA in VAS.

For the between-group analysis, no meaningful effect was noted among the three groups (*p* = 0.562) (Table 3). For the within-group analysis, Figure 2(a) revealed that in relieving the pain with VAS assessment, both LLLA and EA have a significant effect (LLLA: *p* = 0.003; EA: *p* ≤ 0.001); and for pain-reduction amplitude, EA is superior to LA and sham LA with significance (*p* < 0.001, Figure 2(b)).

#### 3.2.2. In Low LF/HF Group (Parasympathetic Tone Predominant or a General Lower Energy Level)

**Figure 3:**
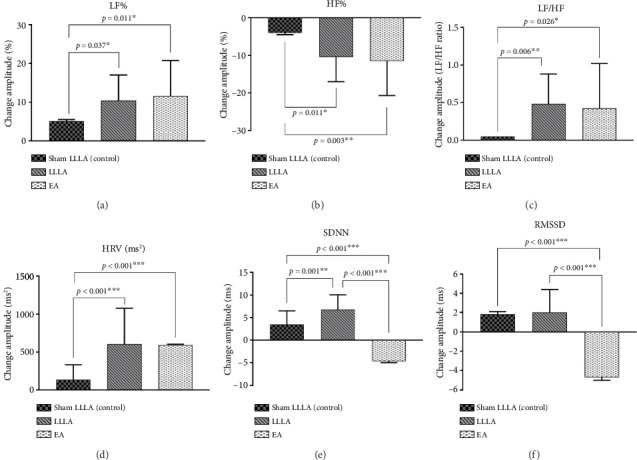
Comparison of the effect of EA and LLLA on HRV in the low LF/HF group (between-group analysis).

In this group (Table 2), there is no significant difference among the three groups over all parameters for the between-group analysis (*p* > 0.05). In within-group analysis, both LLLA and EA benefit patients by balancing autonomic activity. The benefit comes from LLLA and EA adjusting sympathetic tone (LF%) and vagus tone (HF%) reciprocally; in the LLLA group, LF% and HF% changed much more (LF% and HF%: *p* = 0.002) than in the EA group (LF% and HF%: *p* = 0.023), while no differences were seen in sham-controlled LLLA (*p* > 0.05). To elaborate further, significant changes were noted in the autonomic dysfunction improvement amplitude only in the low LF/HF group (parasympathetic tone predominant). Compared with sham LLLA, LLLA and EA both had significant changes in reconciling the autonomic balance and increasing HRV activity, which is present in parameters as LF% (Figure 3(a): LLLA: *p* = 0.037, EA: *p* = 0.011); HF% (Figure 3(b): LLLA: *p* = 0.011; EA: *p* = 0.003); LF/HF (Figure 3(c). LLLA: *p* = 0.006; EA: *p* = 0.026); HRV (Figure 3(d). LLLA: *p* < 0.001; EA: *p* < 0.001), SDNN (Figure 3(e). LLLA: *p* < 0.001; EA: *p* < 0.001); and reverse changes over RMMSD (Figure 3(f)).

#### 3.2.3. In High LF/HF Group (Sympathetic Tone Predominant)

For the between-group analysis, a significant effect was noted over LF% (*p* = 0.046) and HF% (*p* = 0.046) among the three groups (Table 3). For the within-group analysis, Table 2 shows that only LLLA significantly normalized LF/HF through adjusting the LF and HF percentage (LF/HF: *p* = 0.031, LF%: *p* = 0.025, HF%: *p* = 0.025), while EA and sham LLLA did not significantly improve the high LF/HF ratio (*p* > 0.05 in all parameters).

The improvement in the LF/HF ratio was also more significant in LLLA than in EA; LLLA neutralized the LF/HF ratio both in the low LF/HF group (*p* = 0.002) and in the high LF/HF group (*p* = 0.031), yet EA improved only the autonomic imbalance in the low LF/HF group (*p* = 0.036).

**Figure 4:**
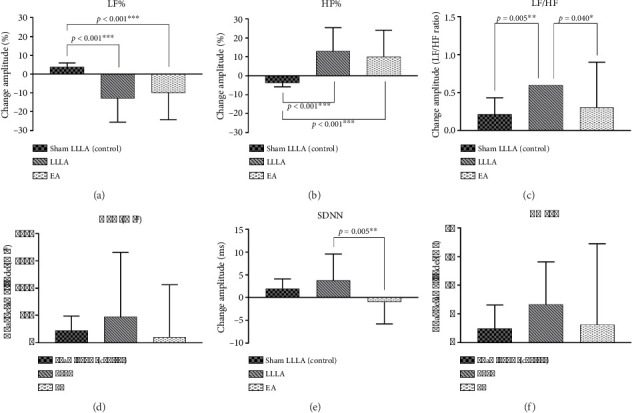
Comparison of the effect of EA and LLLA on HRV in the high LE/HF group (between-group analysis).

More specifically, in the high LF/HF group (sympathetic tone predominant) (Figure 4), LLLA and EA only had significant changes in reconciling the autonomic balance, which was reflected in LF% (Figure 4(a) LLLA: *p* < 0.001; EA: *p* < 0.001); HF% (Figure 4(b) LLLA: *p* = 0.011; EA: *p* = 0.003) and LF/HF (Figure 4(c) LLLA vs. sham: *p* = 0.005; LLLA vs. EA: *p* = 0.040).

To explore potential mechanistic differences when comparing LLLA and EA in autonomic dysfunction, we further used multivariate regression analysis to control confounders, and there is no difference coming from these factors. The data are shown in supporting 4.

### 3.3. Comparison of Effects of EA and LLLT on Symptoms (VMSS) and Quality of Life

For the between-group analysis, no significant effect was noted among the three groups (*p* = 0.751) (Table 3). For within-group analysis, Table 3 shows that both types of acupuncture improved pain, symptoms, and quality of life according to symptom and daily activity evaluation with VMSS (LLLA: *p* = 0.163; EA: *p* < 0.001) and quality-of-life assessment with SF-12 (physical component score [PCS], LLLA: *p* = 0.002; EA: *p* = 0.0.03; mental component score: LLLA: *p* = 0.007; EA *p* < 0.001; and total SF-12 score, LLLA: *p* = 0.019, EA: *p* < 0.001.

**Figure 5:**
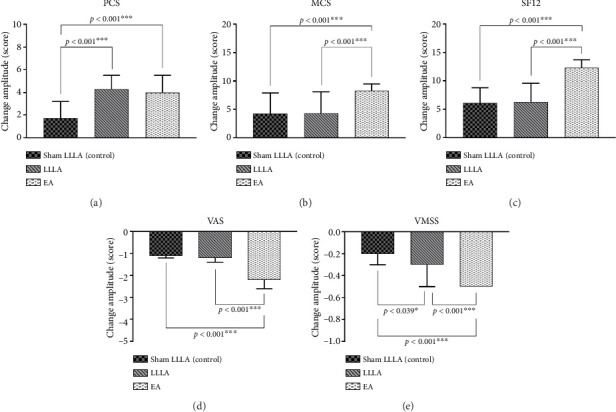
Comparison of the effect of EA and LLLA on symptoms (VAS/VMSS) and quality of life (SF12).

Between-group analysis (Figure 5) showed that EA improved pain (VAS), symptoms (VMSS), and total quality of life (SF-12) significantly more than LLLA (*p* < 0.001).

### 3.4. Comparison of the Effects of EA and LLLA on Cytokine Change

The between-group analysis noted no significant difference among three groups in progesterone (*p* = 0.802) and PGE2 (*p* = 0.350). Furthermore, the intragroup analysis showed no significant differences in progesterone changes before and after the intervention (LLLA: *p* = 0.465; EA: *p* = 0.975); whereas in the PGE2 evaluation, a significant change was only noted in the EA group (EA: *p* = 0.022; LLLA: *p* = 0.072) (Table 3).

**Figure 6:**
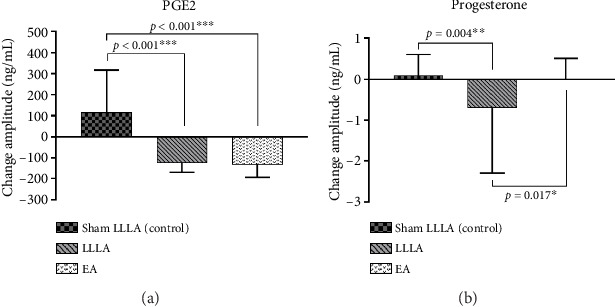
Comparison of the effect of EA and LLLA on cytokine change (progesterone and PGE2).

Between-group analysis showed that both LLLA and EA reduced PGE2 significantly (Figure 6, *p* < 0.001) compared with the sham control, but no significantly different effects were found between LLLA and EA. In progesterone evaluation, only LLLA significantly reduced progesterone compared to EA (*p* = 0.017).

## 4. Discussion

### 4.1. Summary of Main Findings

**Figure 7:**
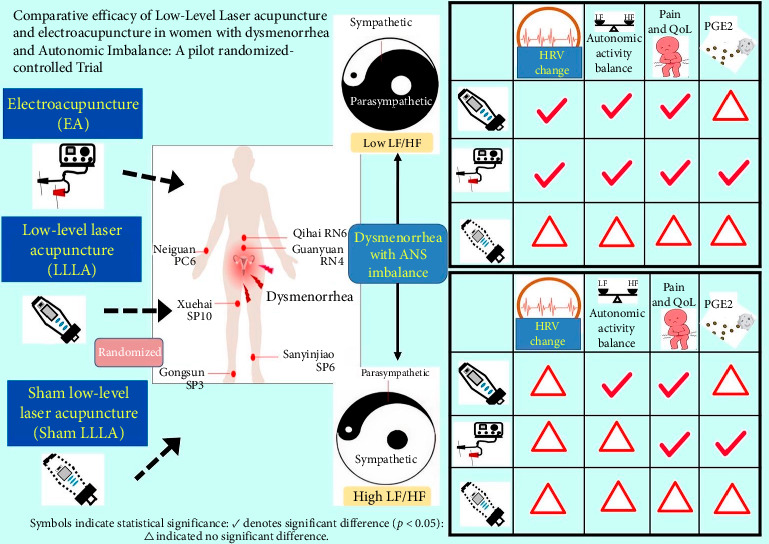
Brief graphic abstract.

By contrast to the sham-controlled acupuncture, both LLLA and EA were significantly effective in pain control, HRV regulation, symptom improvement, and PGE2 reduction. Between-group analysis confirmed that in the low LF/HF group (a relatively lower energy status), more significant benefits were noted no matter if LLLA or EA was used, especially targeting the HRV change and reconciling the autonomic imbalance (LF/HF ratio). These findings suggest that while both modalities are effective, their relative benefits may differ depending on the underlying autonomic profile of the patient.  Figure 7 is a brief graphic abstract for the study.

### 4.2. Different Effects of Acupuncture on HRV Depend on Individual ANS Status (Low or High LF/HF Group)

In the context of TCM, this relationship is often analogized to the interplay between Yin and Yang, where health is defined by their dynamic equilibrium. A lower LF/HF ratio indicates parasympathetic predominance, while a higher ratio suggests sympathetic dominance.

The LF/HF ratio, derived from HRV, serves as a dynamic marker reflecting the balance between sympathetic and parasympathetic activity within the ANS. In the present study, LLLA and EA significantly improved the HRV parameters in the low LF/HF group compared to that in the high LF/HF group (Table 2). In the context of TCM [29, 30], this relationship is often analogized to the interplay between Yin and Yang, where health is defined by their dynamic equilibrium [31, 32]. A lower LF/HF ratio indicates parasympathetic predominance, while a higher ratio suggests sympathetic dominance [33].

ANS imbalance can manifest as a decrease in function (e.g., pure autonomic failure) or an increase in function [34]. Complex interactions between the vagus and sympathetic nervous systems have been simplified to antagonism, emphasizing their opposition yet complementarity to each other [35]. The between-group analysis (Figures 3 and 4) showed that the effect of LLLA and EA is more pronounced in subjects with a low LF/HF ratio, suggesting a state-dependent response to acupuncture. Conversely, in individuals with a high LF/HF ratio, acupuncture primarily induces bidirectional changes in LF% and HF%, contributing to the restoration of autonomic balance, which is compatible with a previous study [15].

The antagonistic yet complementary relationship between the sympathetic and parasympathetic branches of the ANS underlies the therapeutic effects observed. Acupuncture's ability to shift sympathovagal balance toward parasympathetic dominance is supported by systematic reviews and meta-analyses [36, 37]. However, the magnitude and direction of these effects may vary depending on baseline autonomic status and specific intervention parameters. These findings underscore the importance of considering ANS state when evaluating the efficacy of acupuncture for autonomic regulation and highlight the need for further research into subgroup-specific responses and underlying neurophysiological mechanisms.

Acupuncture has demonstrated the capacity to modulate ANS activity, particularly in individuals with autonomic imbalance. In those with elevated LF/HF ratios—indicative of sympathetic dominance—acupuncture, especially LLLA, can normalize the LF/HF ratio, although not all HRV parameters are significantly altered. Conversely, in subjects with low LF/HF ratios, acupuncture enhances overall HRV, increases both HF and LF components, and restores sympathovagal balance. These effects align with TCM theories of regulating Yin–Yang balance and augmenting Qi (energy).

Clinical studies confirm that acupuncture can decrease sympathetic activity and increase parasympathetic tone, as evidenced by reductions in LF/HF ratio and increases in HF power [38]. The therapeutic impact is more pronounced in those with baseline autonomic dysfunction, highlighting the importance of individual ANS status in predicting acupuncture efficacy. Further research is warranted to clarify the mechanisms underlying these state-dependent responses and their clinical relevance in conditions such as dysmenorrhea [39].

### 4.3. LLLA Has More Potential Than EA in Regulating HRV in Low LF/HF Status

As in the present study, previous studies also denoted that both LLLA and EA could increase vagal activity, yet in different disease statuses, patients may have diverse LF/HF patterns and varied responses to acupuncture stimulation [40, 41]. From the perspective of autonomic activity regulation, studies have demonstrated that dysmenorrhea was associated with reduced vagal activity (HF) or autonomic imbalance [42], and our results denoted that both LLLA and EA enhanced vagal activity (HF%) regardless of whether LF/HF status was low or high.

Furthermore, between-group analysis (Figures 3 and 4) showed that LLLA had more significant effects than EA in the low LF/HF group. Therefore, we assume that the laser beam provides extra energy in relatively low-energy status and low-level laser stimulation of acupoints appears to influence peripheral and central nervous system activity [43]. In our study, LLLA's effect is more prominent in a relatively lower energy status (low LF/HF) as for increasing the HRV and reconciling the LF/HF. This finding is compatible with previous studies that LA has a tendency to increase the ratio of parasympathetic and decrease that of sympathetic nervous system activities [44]. Previous studies noted that LLLA induces a photobiomodulatory (PBM) effect on cells and tissues, which contributes to a directed modulation of cell behaviors and increasing electron transport, mitochondrial membrane potential, and ATP production [45]. ATP production is an important issue in maintaining optimal ANS functionality. It is logical that the effect of LLLA is more obvious in lower LF/HF status than high LF/HF status.

### 4.4. Potentially Different Mechanisms of LLLA and EA in Pain Control and Regulating Prostaglandin

The results denote that LLLA has more potential than EA in regulating HRV in low LF/HF status, and EA is more effective in improving dysmenorrhea symptoms and PGE2 reduction. So far we noted the relevant mechanism of LLLA involved promoting the release of endogenous opioids (e.g., endorphins), serotonin, and other neurochemical mediators that contribute to analgesia and relaxation [43]; and reducing the expression of proinflammatory mediators such as inducible nitric oxide synthase (iNOS) and mitogen-activated protein kinases (MAPKs) in the spinal cord, thereby attenuating pain sensitization [46]. The photobiomodulation effects of LLLA at cellular and molecular levels may further regulate the nerve endings, leading to modulation of mitochondrial activity and increased ATP production, which in turn affects cellular signaling and tissue repair [47, 48]. Results of between-group analysis show that both LLLA and EA lead to different levels of reductions in prostaglandin. Whether the different setting or wavelength will affect the LLLA's effect on nerve endings is unknown due to a lack of enough study. However, previous research proved that acupuncture has the effect of anti-inflammation, regardless of whether EA or LLLA is used [49, 50]. Evidence provides insight into the anti-inflammatory effects, neuromodulation, ANS adjustment, and cellular effects of LLLA, although the data remain sparse overall [51, 52]. The LLLA and EA might drive their effect with some overlapping mechanism from meridian theory and some from other aspects at different levels.

## 5. Limitations

This study has several limitations; whether the modes of LLLT, such as wavelength, power output, and energy dose, will affect outcomes may be an issue that needs further investigation. Also, lack of a follow-up phase to determine how long the efficacy can be maintained after performing these two types of acupuncture is a limitation that must be corrected through further comparative study. Moreover, a sham-acupuncture group involving needle insertion at nonacupoint locations or with minimal stimulation was considered; however, clinical feasibility and ethical considerations (e.g., unnecessary needle puncture) made implementation difficult in this patient population. Furthermore, the study focused on noninvasive and minimally invasive interventions, and the main comparator for noninvasiveness was the sham-laser group. This introduces potential for performance and response bias, particularly in self-reported outcomes (e.g., VAS and SF-12), and accordingly encourages future studies to address this limitation.

## 6. Conclusion

This rigorously designed, sham-controlled trial provides robust evidence that both LLLA and EA are effective in the management of dysmenorrhea with autonomic imbalance without placebo effect. The nuanced differences in efficacy across autonomic subgroups underscore the importance of individualized treatment selection. LLLA provides extra energy through photo-bioenergy and provides benefits in regulating HRV, especially in low LF/HF status. Further large-scale, multicenter studies are warranted to confirm these findings and elucidate the mechanisms underlying the differential effects observed between LLLA and EA.

## Figures and Tables

**Table 1:** Demographic characteristics of study participants.

Demographic/groups	Sham LLLA (control) (*N* = 38) mean ± SD	LLLA (*N* = 38) Mean ± SD	EA (*N* = 38) Mean ± SD	*p-*value
Basic data				
Age (years)	36.74 ± 6.43	35.8 ± 8	37.8 ± 6.4	0.461
Body mass index (kg/m^2^)	23.09 ± 3.77	23.4 ± 4.1	23.6 ± 4	0.852
Menstruation related data				
Age at menarche (years)	12.8 ± 1.6	12.7 ± 1.6	13 ± 1.7	0.718
Menstrual cycle duration (days)	28.8 ± 2.2	28.7 ± 2.2	28.7 ± 2.4	0.976
Menstrual irregularity, *n* (%)	5 (14.50%)	4 (11.1%)	3 (9.1%)	0.761
Menstrual pain scores (VAS)	5.5 ± 2.7	5.4 ± 2.8	5.7 ± 2.6	0.886
Severity of dysmenorrhea (VMSS)	1.7 ± 1.0	1.7 ± 1.1	1.8 ± 0.8	0.875
SF-12 quality-of-life scores				
PCS	37.9 ± 8.52	37.94 ± 7.62	37.87 ± 9.34	0.999
MCS	32.98 ± 10.93	30.05 ± 9.48	35.27 ± 11.63	0.109
HRV-related parameters				
SDNN (ms)	39.2 ± 15.7	37.8 ± 14.8	38.6 ± 16.7	0.927
R-MSSD (ms)	36 ± 19.4	32.6 ± 24.3	33.3 ± 19.3	0.761
PNN50 (%)	16.6 ± 21.2	14.8 ± 21.7	13.9 ± 17	0.837
LF (%)	48.4 ± 16.8	50.9 ± 20.4	50.8 ± 20.3	0.814
HF (%)	51.6 ± 16.8	49.2 ± 20.4	49.2 ± 20.3	0.821
LF (ms^2^)	345.3 ± 359.5	351.6 ± 652.1	350.2 ± 463.4	0.998
HF (ms^2^)	438.5 ± 535	405 ± 607.1	358.9 ± 404.8	0.801
LF/HF	1.3 ± 0.8	1.5 ± 1.3	1.4 ± 1.1	0.725
VLF (ms^2^)	932.7 ± 704.2	870.2 ± 831.9	1048.2 ± 957.5	0.832
TP (ms^2^)	1766.5 ± 1443.6	1626.8 ± 1962.7	1757.3 ± 1562.2	0.146

*Note:* All data are shown as mean ± SD. SDNN: standard deviation of adjacent peak-to-peak (NN) intervals. RMMSD: square root of the mean of the sum of the squares of differences between adjacent NN intervals (ms). pNN50: % of differences of adjacent RR intervals > 50 ms. LF (%): low-frequency component normalized. HF (%): high-frequency component normalized. LF: low-frequency component, 0.04–0.15 Hz (ms^2^) (measure of sympathetic function). HF: high-frequency component, 0.15–0.4 Hz (ms^2^) (measure of parasympathetic function). LF/HF: sympathetic nerve/parasympathetic nerve balance. VLF: very-low-frequency band in absolute and normalized values.

Abbreviation: HRV, heart rate variability.

**Table 2:** Comparison the effect of EA, LLLA, and Sham LLLA on HRV in low and high LF/HF group.

**Low LF/HF ratio**	**Sham LLLA (control) (n = 19)**			**LLLA (n = 19)**			**EA (n = 19)**			**△**
	**Mean ± SD**			**Mean ± SD**			**Mean ± SD**			
	**Baseline**	**3-month treatment**	**p**	**Baseline**	**3-month treatment**	**p**	**Baseline**	**3-month treatment**	**p**	**Interaction ANOVA p**
	**(n = 19)**	**(n = 19)**		**(n = 19)**	**(n = 19)**		**(n = 19)**	**(n = 19)**		

SDNN (ms)	37.05 ± 12.78	40.51 ± 15.83	0.463	39.1 ± 16.3	45.9 ± 19.6	0.118	43.9 ± 16	39.2 ± 15.7	0.132	0.325
RMSSD	42.68 ± 18.28	44.49 ± 18.55	0.764	43.4 ± 19	45.4 ± 21.4	0.453	43 ± 20.8	38 ± 19.4	0.182	0.702
PNN50	24.56 ± 19.15	26.82 ± 18.19	0.711	24.6 ± 20	28.8 ± 22.1	0.255	22.8 ± 19.7	19.6 ± 21.2	0.649	0.735
LF (%)	33.55 ± 19.32	38.61 ± 18.9	0.420	33.4 ± 7.9	43.8 ± 14.5	0.002^∗∗^	30.8 ± 7.6	42.4 ± 16.8	0.023^∗^	0.645
HF (%)	66.45 ± 19.32	62.39 ± 18.9	0.517	66.6 ± 7.9	56.2 ± 14.5	0.002^∗∗^	69.2 ± 7.6	57.7 ± 16.8	0.023^∗^	0.559
LF (ms^2^)	297.67 ± 247.6	367.02 ± 429.06	0.546	290.3 ± 394.8	568.4 ± 753.2	0.022^∗^	261 ± 188	345.3 ± 359.5	0.532	0.723
HF (ms^2^)	554.4 ± 384.78	578.08 ± 431.09	0.859	583.6 ± 520.2	627 ± 532.8	0.523	532.8 ± 464.5	488.5 ± 535	0.427	0.925
LF/HF	0.59 ± 0.2	0.64 ± 0.2	0.446	0.5 ± 0.2	0.98 ± 0.6	0.002^∗∗^	0.5 ± 0.2	0.92 ± 0.8	0.036^∗^	0.085
VLF (ms^2^)	894.7 ± 631.5	1277.3 ± 1125.3	0.204	874.85 ± 622.47	1105.84 ± 820.78	0.097	932.7 ± 704.2	1397.7 ± 1141	0.069	0.107
HRV (ms^2^)	1874 ± 1300.1	2005 ± 2100.2	0.841	1784.9 ± 2049	2389.1 ± 2521.1	< 0.001^∗∗∗^	1945.1 ± 2505.2	2537.8 ± 2494.4	0.003^∗∗^	0.887

**High LF/HF ratio**	**Sham LLLA (control) (n = 19)** **Mean ± SD**			**LLLA (n = 19)** **Mean ± SD**			**EA (n = 19)** **Mean ± SD**			**△**
	**Baseline**	**3-month treatment**	**p**	**Baseline**	**3-month treatment**	**p**	**Baseline**	**3-month treatment**	**p**	**Interaction ANOVA p**
	**(n = 19)**	**(n = 18)**		**(n = 18)**	**(n = 18)**		**(n = 19)**	**(n = 19)**		

SDNN (ms)	34.27 ± 15.84	36.26 ± 13.71	0.681	33.7 ± 15.9	37.5 ± 21.7	0.492	33.9 ± 16.4	32.9 ± 21.2	0.962	0.837
RMSSD	23.66 ± 18.63	26.12 ± 14.52	0.653	26.7 ± 18.2	33.4 ± 25.6	0.214	24.8 ± 13.4	27.9 ± 27.6	0.962	0.887
PNN50	6.5 ± 5.8	10.26 ± 19.71	0.453	7.4 ± 12.7	12.7 ± 20	0.162	6.1 ± 9	10.5 ± 21.8	0.909	0.979
LF (%)	62.51 ± 17.28	66.37 ± 15.19	0.469	64.7 ± 7.4	51.7 ± 19.9	0.025^∗^	68.4 ± 6.7	58.4 ± 20.8	0.149	0.046^∗^
HF (%)	37.49 ± 17.28	33.63 ± 15.19	0.469	35.3 ± 7.4	48.3 ± 19.9	0.025^∗^	31.6 ± 6.7	41.6 ± 20.8	0.149	0.046^∗^
LF (ms^2^)	388.63 ± 407.07	386.18 ± 218.75	0.982	335.3 ± 462.2	319.9 ± 484.8	0.334	454.4 ± 600.4	357.2 ± 843	0.149	0.917
HF (ms^2^)	190.52 ± 402.33	248.55 ± 313.15	0.623	182.6 ± 226.9	383.2 ± 620.9	0.171	205.5 ± 274.4	331.3 ± 671.9	0.836	0.790
LF/HF	2.03 ± 1.1	1.82 ± 0.88	0.520	2 ± 0.9	1.4 ± 0.9	0.031^∗^	2.3 ± 0.8	2 ± 1.4	0.381	0.683
VLF (ms^2^)	833.8 ± 659.55	1044.96 ± 680.1	0.338	856.2 ± 802.9	1146 ± 1600.4	0.872	739.8 ± 649.2	815.1 ± 948.7	0.723	0.884
HRV (ms^2^)	1506.44 ± 1308.99	1728.21 ± 1043.2	0.567	1374.1 ± 1417.5	1849.1 ± 2602.6	0.687	1399.7 ± 1410.6	1503.6 ± 2367.7	0.868	0.899

*Note:* All data are shown as mean ± SD. SDNN: standard deviation of adjacent peak-to-peak (NN) intervals. RMMSD: square root of the mean of the sum of the squares of differences between adjacent NN intervals (ms). pNN50: % of differences of adjacent RR intervals > 50 ms. LF (%): low-frequency component normalized. HF (%): high-frequency component normalized. LF: low-frequency component, 0.04–0.15 Hz (ms^2^) (measure of sympathetic function). HF: high-frequency component, 0.15–0.4 Hz (ms^2^) (measure of parasympathetic function). LF/HF: sympathetic nerve/parasympathetic nerve balance. VLF: very-low-frequency band in absolute and normalized values.

Abbreviation: HRV, heart rate variability.

^∗^ *p* < 0.05 with statistical significance.

^∗∗^ *p* < 0.01 with statistical significance.

^∗∗∗^ *p* < 0.001 with statistical significance.

**Table 3:** Comparison of the effect of EA, LLLA, and Sham LLLA on symptoms (VAS/VMSS), quality of life (SF12), and cytokine change (progesterone and PGE2).

**VAS** **VMSS** **SF-12 PCS** **MCS/groups**	**Sham LLLA (control) (n = 38)**			**LLLA (n = 38)**			**EA (n = 38)**			**△**
	**Mean ± SD**			**Mean ± SD**			**Mean ± SD**			
	**Baseline**	**3-month treatment**	**p**	**Baseline**	**3-month treatment**	**p**	**Baseline**	**3-month treatment**	**p**	**Interaction ANOVA p**

VAS	5.3 ± 2.7	4.2 ± 2.6	0.186	5.4 ± 2.8	4.2 ± 2.6	0.003^∗∗^	5.7 ± 2.6	3.5 ± 2.2	< 0.001^∗∗∗^	0.562
VMSS	1.7 ± 1.0	1.5 ± 0.9	0.500	1.7 ± 1.1	1.4 ± 0.9	0.163	1.8 ± 0.8	1.3 ± 0.8	< 0.001^∗∗∗^	0.751
SF12	77.6 ± 18.3	83.7 ±0 .15.6	0.252	77.4 ± 19	83.7 ± 15.7	0.019^∗^	75.9 ± 17.8	88.2 ± 16.4	< 0.001^∗∗∗^	0.644
PCS	42.2 ± 7.6	43.9 ± 9.1	0.515	40.1 ± 10.0	44.4 ± 8.8	0.002^∗∗^	40.1 ± 10.4	44.1 ± 8.9	0.003^∗∗^	0.778
MCS	35.4 ± 13.8	39.6 ± 10.1	0.267	35.5 ± 13.6	39.8 ± 9.8	0.007^∗^	35.8 ± 12.1	44.1 ± 10.9	< 0.001^∗∗∗^	0.664

**Cytokine/groups**	**Sham LLLA (control) (n = 38)** **Mean ± SD**			**LLLA (n = 38)** **Mean ± SD**			**EA (n = 38)** **Mean ± SD**			**△**
	**Baseline**	**3-monthtreatment**	**p**	**Baseline**	**3-month treatment**	**p**	**Baseline**	**3-month treatment**	**p**	**Interaction ANOVA p**

Progesterone (ng/mL)	3.6 ± 3.6	3.7 ± 4.1	0.914	3.5 ± 4.6	2.8 ± 3	0.465	3.5 ± 3.5	3.5 ± 4	0.975	0.802
PGE2 (pg/mL)	657.4 ± 579.4	772.1 ± 781.9	0.488	736 ± 462	610.5 ± 506.4	0.072	755 ± 476.2	621.1 ± 536.5	0.022^∗^	0.350

*Note:* All data are shown as mean ± SD. SF-12: quality-of-life assessment; PGE2, prostaglandin E2.

Abbreviations: EA, electroacupuncture; LLLA, low-level laser acupuncture; MCS, mental component score; PCS, physical component score; VAS, visual analogue scale; VMSS: verbal multidimensional scoring system.

^∗^ *p* < 0.05 with statistical significance.

^∗∗^ *p* < 0.01 with statistical significance.

^∗∗∗^ *p* < 0.001 with statistical significance.

## Data Availability

The data that support the findings of this study are available on request from the corresponding author. The data are not publicly available due to privacy or ethical restrictions.

## Nomenclature

EA: Electroacupuncture
LLLA: Low-level laser acupuncture
SDNN: Standard deviation of adjacent peak-to-peak (NN) intervals
RMMSD: Square root of the mean of the sum of the squares of differences between adjacent NN intervals (ms)
pNN50: % of differences of adjacent RR intervals > 50 ms
LF (%): Low-frequency component normalized
HF (%): High-frequency component normalized
LF: Low-frequency component, 0.04–0.15 Hz (ms^2^) (measure of sympathetic function)
HF: High-frequency component, 0.15–0.4 Hz (ms^2^) (measure of parasympathetic function)
LF/HF: Sympathetic nerve/parasympathetic nerve balance
VLF: Very-low-frequency band in absolute and normalized values
HRV: Heart rate variability
VAS: Visual analogue scale
VMSS: Verbal multidimensional scoring system
SF-12: Quality-of-life assessment
PCS: Physical component score
MCS: Mental component score

## Supporting Information

Supporting Information 1 Supporting 1: The equipment of laser acupuncture.

Supporting Information 2 Supporting 2: The acupoints applied in the study.

Supporting Information 3 Supporting 3: The study scheme with timeline.

Supporting Information 4 Supporting 4: Multivariable linear regression analyses of the effect of confounders among different groups of acupuncture intervention in three groups.
