# Supplementary figures and images for: Comparative Efficacy of Low-Level Laser Acupuncture and Electroacupuncture in Women With Dysmenorrhea and Autonomic Imbalance: A Pilot Randomized-Controlled Trial
Source: Pain Res Manag. 2025 Oct 23;2025:3494216. doi: 10.1155/prm/3494216 (PMC12575050; doi:10.1155/prm/3494216)

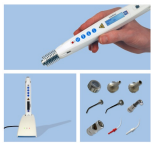

Supplement: Supporting Information 1 — Supporting 1: The equipment of laser acupuncture. [file 3494216.f1.pdf]

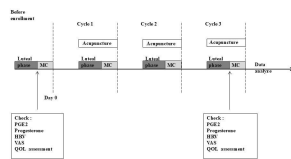

Supplement 3 study scheme

Supplement: Supporting Information 3 — Supporting 3: The study scheme with timeline. [file 3494216.f3.pdf]
