# Supplementary material for: Comparative Efficacy of Low-Level Laser Acupuncture and Electroacupuncture in Women With Dysmenorrhea and Autonomic Imbalance: A Pilot Randomized-Controlled Trial
Source: Pain Res Manag. 2025 Oct 23;2025:3494216. doi: 10.1155/prm/3494216 (PMC12575050; doi:10.1155/prm/3494216)
Supplement: Supporting Information 2 — Supporting 2: The acupoints applied in the study. [file 3494216.f2.pdf]

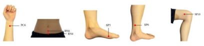

Acupoints	Location
PC6 (Inner Pass)内关	2 cun above the wrist crease between the tendons of palmaris longus and flexor carpi radialis.
RN4 (Origin Pass)关元	2 cun above CV 2 (pubic symphysis).
RN6 (Sea of Qi)气海	Midway between CV 5 and CV 7, 1.5 cun below CV 8 (umbilicus).
SP3 (Yellow Emperor)公孙	In a depression distal and inferior to the base of the 1st metatarsal bone at the junction of the red and white skin.
SP6 (Three Yin Intersection)三阴交	3 cun directly above the tip of the medial malleoulus on the posterior border of the tibia.
SP10 (Sea of Blood)血海	With knee flexed, 2 cun above the superior medial border of the patella on the bulge of the medial portion of quadriceps femoris (vastus medialis).
