# Supplementary material for: Comparative Efficacy of Low-Level Laser Acupuncture and Electroacupuncture in Women With Dysmenorrhea and Autonomic Imbalance: A Pilot Randomized-Controlled Trial
Source: Pain Res Manag. 2025 Oct 23;2025:3494216. doi: 10.1155/prm/3494216 (PMC12575050; doi:10.1155/prm/3494216)
Supplement: Supporting Information 4 — Supporting 4: Multivariable linear regression analyses of the effect of confounders among different groups of acupuncture intervention in three groups. [file 3494216.f4.docx]

| **Autonomic nervous parameters** | **Low**  **LF/HF ratio** | **Sham LLLA (Control) (n=19)** | | **LLLA(n=19)** | | **EA(n=19)** | |
| --- | --- | --- | --- | --- | --- | --- | --- |
|  | **confounders** | **coefficient** | ***P*** | **coefficient** | ***p*** | **coefficient** | ***p*** |
| **HRV** | **Age** | -0.320 | 0.565 | -0.498 | 0.184 | -1.179 | 0.163 |
|  | BMI | -0.024 | 0.975 | -0.327 | 0.538 | -0.037 | 0.975 |
|  | **VAS** | -0.808 | 0.785 | -3.683 | 0.063 | -0.815 | 0.883 |
|  | VMSS | 4.952 | 0.559 | 4.054 | 0.430 | -5.782 | 0.744 |
| **LF/HF** | **Age** | -0.559 | 0.470 | -0.641 | 0.252 | -0.056 | 0.113 |
|  | **BMI** | -0.077 | 0.941 | -0.370 | 0.641 | 0.080 | 0.128 |
|  | **VAS** | -1.479 | 0.719 | -5.848 | 0.051* | -0.271 | 0.250 |
|  | **VMSS** | 7.238 | 0.539 | 8.623 | 0.268 | 1.747 | 0.037* |
| **HF%** | **Age** | -0.478 | 0.493 | -0.591 | 0.227 | 1.286 | 0.065 |
|  | **BMI** | -0.058 | 0.950 | -0.357 | 0.607 | -1.880 | 0.073 |
|  | **VAS** | -1.254 | 0.735 | -5.110 | 0.050* | 3.127 | 0.478 |
|  | **VMSS** | 6.472 | 0.542 | 7.062 | 0.297 | -29.47 | 0.059 |
| **LF%** | **Age** | -0.395 | 0.527 | -0.544 | 0.201 | -0.302 | 0.425 |
|  | **BMI** | -0.041 | 0.960 | -0.341 | 0.571 | 1.180 | 0.063 |
|  | **VAS** | -1.018 | 0.759 | -4.359 | 0.054 | 1.282 | 0.474 |
|  | **VMSS** | 5.681 | 0.550 | 5.454 | 0.352 | -0.722 | 0.877 |
| **RMMSD** | **Age** | -0.095 | 0.870 | -0.438 | 0.379 | -0.789 | 0.427 |
|  | **BMI** | -0.851 | 0.279 | -0.256 | 0.719 | 0.273 | 0.0853 |
|  | **VAS** | -3.187 | 0.307 | -4.075 | 0.121 | -0.019 | 0.998 |
|  | **VMSS** | 4.853 | 0.583 | 4.153 | 0.547 | -2.539 | 0.357 |
| **Total Power** | **Age** | -1.203 | 0.146 | -0.822 | 0.240 | 0.929 | 0.463 |
|  | **BMI** | 0.084 | 0.938 | 0.617 | 0.534 | 0.123 | 0.755 |
|  | **VAS** | 0.413 | 0.923 | -8.089 | 0.032* | 0.793 | 0.727 |
|  | **VMSS** | 5.123 | 0.677 | 12.392 | 0.204 | 0.891 | 0.265 |

**Multivariable linear regression analyses of the effect of confounders among different group of acupuncture intervention in**

| **Autonomic nervous parameters** | **High**  **LF/HF ratio** | **Sham LLLA (Control) (n=19)** | | **LLLA(n=19)** | | **EA(n=19)** | |
| --- | --- | --- | --- | --- | --- | --- | --- |
|  | **confounders** | **coefficient** | ***P*** | **coefficient** | ***p*** | **coefficient** | ***p*** |
| **HRV** | **Age** | 0.035 | 0.967 | 0.519 | 0.193 | -0.498 | 0.184 |
|  | BMI | 2.159 | 0.074 | 0.040 | 0.915 | -0.327 | 0.538 |
|  | **VAS** | 1.651 | 0.694 | 3.249 | 0.045 | -3.683 | 0.063 |
|  | VMSS | -5.931 | 0.434 | -5.644 | 0.127 | 4.054 | 0.430 |
| **LF/HF** | **Age** | -0.109 | 0.923 | -0.101 | 0.500 | -0.641 | 0.252 |
|  | **BMI** | 3.359 | 0.043 | 0.199 | 0.322 | -0.370 | 0.641 |
|  | **VAS** | 2.942 | 0.660 | -0.232 | 0.555 | -5.848 | 0.051 |
|  | **VMSS** | -7.271 | 0.470 | 1.098 | 0.397 | 8.623 | 0.268- |
| **HF%** | **Age** | -0.056 | 0.956 | 2.259 | 0.457 | -0.591 | 0.227 |
|  | **BMI** | 2.946 | 0.050 | -3.184 | 0.405 | -0.357 | 0.607 |
|  | **VAS** | 2.487 | 0.627 | 2.975 | 0.696 | -5.110 | 0.050 |
|  | **VMSS** | -6.841 | 0.458 | -13.441 | 0.580 | 7.062 | 0.297 |
| **LF%** | **Age** | -0.005 | 0.976 | -2.322 | 0.440 | -0.544 | 0.201 |
|  | **BMI** | 2.532 | 0.060 | 3.089 | 0.410 | -0.341 | 0.571 |
|  | **VAS** | 2.054 | 0.658 | -2.814 | 0.706 | -4.359 | 0.054 |
|  | **VMSS** | -6.392 | 0.444 | 12.815 | 0.590 | 7.454 | 0.352 |
| **RMMSD** | **Age** | -0.307 | 0.712 | -0.285 | 0.754 | -0.438 | 0.379 |
|  | **BMI** | 2.668 | 0.031 | -0.503 | 0.658 | -0.256 | 0.719 |
|  | **VAS** | 7.709 | 0.083 | 3.4587 | 0.242 | -4.075 | 0.121 |
|  | **VMSS** | -4.470 | 0.543 | -8.825 | 0.313 | 4.153 | 0.547 |
| **Total Power** | **Age** | 0.467 | 0.759 | -4.269 | 0.486 | -0.822 | 0.240 |
|  | **BMI** | 3.124 | 0.137 | 4.542 | 0.734 | 0.617 | 0.534 |
|  | **VAS** | -3.332 | 0.658 | 2.180 | 0.250 | -8.089 | 0.032 |
|  | **VMSS** | -10.384 | 0.444 | -39.39 | 0.451 | 12.392 | 0.204 |
